# The Incidence of Hypogonadotropic Hypogonadism in Type 2 Diabetic Men in Polish Population

**DOI:** 10.1155/2013/767496

**Published:** 2013-10-10

**Authors:** Michał Rabijewski, Lucyna Papierska, Wojciech Zgliczyński, Paweł Piątkiewicz

**Affiliations:** ^1^Department of Internal Diseases, Diabetology and Endocrinology, Warsaw Medical University, Kondratowicza Street 8, 03-242 Warsaw, Poland; ^2^Department of Endocrinology, Medical Centre for Postgraduate Education, Warsaw, Poland

## Abstract

The aim of this study was to investigate the incidence of hypogonadotropic hypogonadism (HH) in type 2 diabetic men (T2DM) in population of Polish men and examine the possible influence of estradiol levels and glycemic control. We evaluated TT, cfT, estradiol, and glycemic control (HbA1c) in 184 diabetic men and in 149 nondiabetic control group. The mean HbA1c was 8.6 ± 0.2% and 6.1 ± 0.3% and cfT concentration was 0.315 ± 0.08 nmol/L and 0.382 ± 0.07 nmol/L, respectively. T2DM had higher E2 concentration than nonobese control men (29.4 ± 3.7 pg/mL versus 24.5 ± 2.9 pg/mL). Forty-six percent of T2DM were hypogonadal and 93% had HH. We observed inverse relationship between BMI and cfT (*r* = −0.341, *P* < 0.01) and positive between BMI and E2 (*r* = 0.329, *P* < 0.01). E2 concentration was higher in T2DM with HH versus T2DM with normal TT/cfT concentration (34.5 ± 5.2 versus 27.4 ± 3.4 pg/mL). We observed negative correlation between HbA1c and cfT (*r* = −0.336, *P* < 0.005) but positive between HbA1c and E2 levels (*r* = 0.337, *P* < 0.002). The prevalence of obesity, hypertension, and CVD was higher in men with hypogonadism. High incidence of hypogonadotropic hypogonadism in type 2 diabetic men in Polish population is associated with poor glycemic control and can be secondary to an increase in estradiol concentrations.

## 1. Introduction

The prevalence of diabetes mellitus is rapidly increasing. By the year 2030, there are expected to be almost twice as many older persons with diabetes in developing countries compared to the more developed ones [1]. Incidence of diabetes in Poland is higher than observed in Western European countries. In the general Polish population aged 20–74 years diabetes was diagnosed in 6.8% individuals, including 7.4% men and 6.2% women. Incidence of diabetes increases with age: in men from 0.7% in those aged 20–29 years to 16.3% in those aged >60 years [2]. In Polish-Norwegian Study (PONS) performed among people aged 45–64 diabetes was diagnosed in 12.2% males and 6.4% females [3]. 

Type 2 diabetes in men is associated with lower total testosterone (TT) and free testosterone (fT) levels in cross-sectional studies, and the majority of these men have signs and symptoms of hypogonadism such as erectile dysfunctions (ED), low libido, fatigue, sarcopenia, and depression [4]. The origin of these findings is complex, but about 25–40% type 2 diabetic men (T2DM) have low testosterone concentrations in association with inappropriately low or normal LH and FSH concentrations and diagnosis of hypogonadotropic hypogonadism (HH) can be established. About 4% of T2DM have subnormal testosterone concentrations with elevated LH and FSH concentrations, which can be associated with primary testis dysfunctions [4].

The possible pathophysiological mechanisms underlying HH in T2DM are still unknown. Low testosterone levels in men are associated with insulin resistance and reduced insulin sensitivity [5]. Moreover, low testosterone levels have also been found to predict insulin resistance and the future development of type 2 diabetes [6]. In study of a large number of obese men (mean age 60 years) 44% of T2DM and 33% of age-matched nondiabetic men had subnormal testosterone concentrations [7]. Testosterone levels are inversely related to body mass index (BMI), but the testosterone deficiency in T2DM was not dependent upon obesity, because 25% of nonobese patients also had HH [8].

HH is relatively rare in type 1 diabetes and therefore is not a function of diabetes or hyperglycemia itself [9]. Thus, in view of the inverse relationship between BMI and testosterone concentrations in both type 1 and type 2 diabetes, HH is probably related to insulin resistance [4, 5]. Testosterone can be converted to estradiol in the adipose tissue, and it has been suggested that excessive estrogen secretion in the obese may suppress secretion of GnRH and LH [10]. However, in recent studies estradiol levels in men with HH were significantly lower than in those without HH [11].

Low testosterone concentration in T2DM is associated with two to three times elevated risk of cardiovascular events and death [12]; therefore, serum testosterone should be measured in men with type 2 diabetes mellitus with symptoms suggestive of testosterone deficiency [13].

The high prevalence of diabetes mellitus in population of Polish men is well documented, but the prevalence of HH in T2DM is now unknown. In our earlier study we showed that in a cohort of Polish men with poor health status the prevalence of testosterone deficiency in elderly men with ED is higher than in other countries [14]. So, we hypothesize that the incidence of HH in T2DM also can be higher than in other studies and is associated with late identification of patients with type 2 diabetes and improper glycemic control.

Therefore, the aim of this study was to investigate the incidence of hypogonadotropic hypogonadism in T2DM in Polish population and examine the possible influence of estradiol levels and glycemic control.

## 2. Material and Methods

This was a cross-sectional study of 184 type 2 diabetic men, aged >45 years (age range 47–63 years), who were registered with the Department of Endocrinology, Medical Centre of Postgraduate Education, Warsaw, Poland. All patients gave written informed consent, and the local research ethics committee approved the protocol (CMKP/501-2-1-07-21/2009). 

Patients with known history of hypogonadism, panhypopituitarism, chronic debilitating disease, or were already receiving hormone replacement therapy were excluded from the study. Demographic parameters, clinical history including the duration of diabetes, medications, and the presence of erectile dysfunction and coronary artery disease were collected, and height, weight, fasting glucose, and HbA1c were measured. Type 2 diabetes was diagnosed according to WHO criteria [15]. Nine patients were treated with diet alone, 77 with insulin, and 98 with oral hypoglycemic agents. Height and weight were measured, and BMI was calculated. Cardiovascular disease was defined as self-reported coronary artery disease, cerebrovascular disease, congestive heart failure, or arrhythmia. 

Fasting blood samples were then obtained between 8:00 and 10:00 A.M. to measure serum total testosterone (TT), estradiol (E2), sex hormone binding globuline (SHBG), luteinizing hormone (LH), follicle-stimulating hormone (FSH), prolactin (PRL), fasting plasma glucose (FPG), and HbA1c. All men had their total testosterone (TT), LH, FSH, and PRL levels checked at least once. TT, LH, FSH, and PRL were measured by chemiluminescent immunometric assays (Immulite 2000; DPC USA and Coat-a-Coat; Siemens USA). The normal value for testosterone was 8–28 nmol/L (sensitivity—4 ng/dL), for LH: 2–6 mIU/L (sensitivity—0.05 mIU/L), for FSH: 3–10 mIU/L (sensitivity—0.1 mIU/L), and for PRL: 12–24 ng/mL (sensitivity—0.16 ng/mL). Calculated free testosterone (cFT) was calculated from SHBG, serum albumin, and TT using the method of Vermeulen and colleagues [16]. A free testosterone level <0.255 nmol/L was taken as low. TT concentration <8 nmol/L was considered to be low, and between 8 and 12 nmol/L was considered to be borderline low. HH was defined as TT levels <12 nmol/L LH levels <6 mIU/mL, and FSH levels <8 mIU/mL. Primary hypogonadism was defined as TT levels <12 nmol/L, LH levels >6 mIU/mL, and FSH levels >8 mIU/mL.

Sexual function was assessed according to the International Index of Erectile Function (IIEF-5) questionnaire. Possible scores on the IIEF-5 are 1 to 25 and erectile dysfunction was classified into 5 categories based on the scores, namely, severe—1 to 7, moderate—8 to 11, mild to moderate—12 to 16, mild—17 to 21 and none—22 to 25.

Statistical analysis was performed using statistica software. Data are presented as mean ± SE. Mann Whitney rank sum test was used to compare nonparametric data, and Student's *t* test was used to compare parametric data. Spearman correlation (for nonparametric data) or Pearson correlation (for parametric data) was used to establish correlations. All relationships were assessed by linear univariate and multivariate regression analysis to reduce bias in a cross-sectional study. Results were considered statistically significant at *P* < 0.05.

## 3. Results

 A total of 184 T2DM and 149 nondiabetic control men were evaluated in the study. Characteristics of all T2DM men, patients with HH, primary hypogonadism, normal testosterone concentration, and nonobese control group are shown in Table 1. The mean age in T2DM group and nonobese control group was 58.5 ± 2.3 years and 59.6 ± 3.2 years; mean body mass index (BMI) was 31.4 ± 0.5 kg/m^2^ and 27.6 ± 1.2 kg/m^2^, and mean HbA1c was 8.6 ± 0.2% and 6.1 ± 0.3%, respectively. The mean TT concentration was 13.3 ± 1.65 nmol/L and 17.1 ± 1.7 nmol/L, and cfT concentration was 0.315 ± 0.08 nmol/L and 0.382 ± 0.07 nmol/L. We observed statistically higher E2 concentration in T2DM group than in nonobese control men (29.4 ± 3.7 pg/mL and 24.5 ± 2.9 pg/mL, *P* < 0.001). 

**Figure 1:**
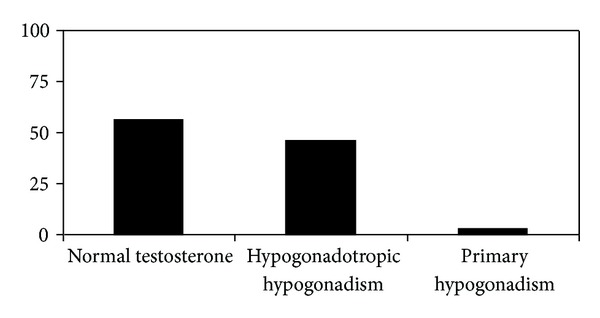
The incidence percentage of normal testosterone levels compared with patients with HH and primary hypogonadism in T2DM in Polish population.

Thus, we showed that TT and cfT concentrations in all T2DM group were statistically significantly lower than in nondiabetic control group with the same average age (Table 1). Forty-six percent of T2DM (*n* = 86) were hypogonadal, and in 82 of these patients (93%) LH and FSH levels were significantly lower when compared with patients with normal TT/cfT levels (3.5 ± 0.3 versus 7.3 ± 0.3 mIU/mL for LH and 5.8 ± 0.6 versus 9.2 ± 0.45 mIU/mL for FSH; *P* < 0.02), and only 4 men had testosterone, LH, and FSH concentrations characteristic to primary hypogonadism.  Figure 1 shows the percentage of patients with normal TT/cfT levels and patients with HH and primary hypogonadism.

Pearson coefficients of BMI and TT concentration showed a statistically significant inverse relationship (*r* = −0.362; *P* < 0.01). Also inverse relationship between BMI and cfT concentration (*r* = −0.341, *P* < 0.01) was observed. There was also positive correlation between BMI and E2 (*r* = 0.329; *P* < 0.01). SHBG correlated inversely with BMI (*r* = −0.277; *P* < 0.05) but positively with age (*r* = 0.538; *P* < 0.001) and TT (*r* = 0.543; *P* < 0.001). E2 concentration was significant higher in T2DM with HH versus T2DM with normal TT/cfT concentration (34.5 ± 5.2 versus 27.4 ± 3.4 pg/mL, resp.), but we did not observed statistical significant differences between T2DM with normal TT/cfT concentration and nonobese control men (27.4 ± 3.4 versus 28.2 ± 3.2 pg/mL, resp.). These results were significant after adjustment for BMI and age.

We observed statistically significant negative correlation between HbA1c and TT and between FPG and TT concentrations (*r* = −0.346, *P* < 0.002 and *r* = −0.345, *P* < 0.002, resp.) and significant negative correlation between HbA1c and cfT concentrations (*r* = −0.336; *P* < 0.005) but there was no correlation between FPG and cfT concentrations. There was also positive correlation between HbA1c and BMI and between HbA1c and E2 levels (*r* = 0.382, *P* < 0.02 and *r* = 0.337; *P* < 0.002, resp.). After adjusting for age, TT/E2 ratio was negatively associated with BMI (*P* < 0.001). 

Multivariate linear regression analysis showed that obesity negatively correlated with TT levels with a mean decrease of 25 ng/dL per 1 kg/m^2^ increase in BMI score (*P* < 0.005). Also, age negatively correlated with TT and cfT levels with a mean decrease of 1.8 ng/dL and 0.003 nmol/L for each year of additional age over 45 years old, respectively (*P* < 0.002).

The degree of erectile dysfunction in all T2DM group was mild in 24% of cases, mild to moderate in 31.5%, moderate in 24.5%, and severe in 20% (Table 2), and there were significant differences between patients with normal TT/cfT concentrations and patients with hypogonadism (*P* < 0.02). IIEF-5 score and TT showed a statistically inverse relationship (*r* = −0.3149, *P* < 0.05).

In Table 3, we presented prevalence of tobacco use, hypertension, dyslipidemia, obesity, and cardiovascular disease (CVD) in all T2DM. The prevalence of each condition, except tobacco use, was above 60% and prevalence of obesity, hypertension, and CVD was significantly higher in men with hypogonadism compared with eugonadal patients. The most common metabolic disorders were obesity (79% in men with hypogonadism and 93% in men with normal TT/cfT levels) and hyperlipidemia (51% and 93%, resp.).

## 4. Discussion

We evaluated the incidence of HH in Polish population of 184 T2DM. It was the first study performed in the relative large population of men in Poland. To our knowledge, this is the first report showing such high incidence of HH in population of T2DM with poor health status. In our cohort 46% patients were hypogonadal and 93% of these patients covered criteria for recognizing of HH. 

Dhindsa et al. [8] described the association of HH with type 2 diabetes in 103 T2DM in mean age 54.7 years. Authors revealed that 33% of studied T2DM were hypogonadal, and LH and FSH levels were significantly lower in the hypogonadal group compared with patients with normal testosterone levels. In study of Rhoden et al. [17] in 116 diabetic men TT serum levels were subnormal in 34% of patients, and TT levels were strongly associated with elevated BMI. Corona et al. [18] in an investigation of 1200 patients with ED (16% with type 2 diabetes) observed HH in 24.5% diabetic men versus 12.6% in the rest of the group. Differences in the prevalence of hypogonadism retained significance after adjustment for age and BMI. In cross-sectional study of 355 type 2 diabetic men aged >30 years Kapoor et al. [19] observed low testosterone levels in diabetic men, and a significant proportion of these men had symptoms of hypogonadism. In a cross-sectional survey of 580 men with type 2 diabetes Laaksonen et al. [20] found that 43% of men with type 2 diabetes had a reduced TT concentration, and low testosterone levels were independently associated with insulin resistance.

The incidence of hypogonadism among patients with type 2 diabetes was significantly greater in our study than observed in studies cited above. Hypogonadism can be associated with diabetes *per se*, but vascular factors, drugs, tobacco, alcohol, and systemic diseases such as hypertension, heart diseases, and dyslipidemia can be risk factors of testosterone deficiency. In our study we observed high prevalence of tobacco use, hypertension, dyslipidemia, cardiovascular disease, and obesity (over 60% for each condition). Very high prevalence of these diseases, higher than in cited studies, can be one of the explanation of differences in incidence of hypogonadism between population of Polish men and patients in Western Europe countries and the United States. In our earlier publication we showed [14] that late-onset hypogonadism (LOH) was very common in population of Polish men presenting ED and correlated negatively with age, obesity, and dyslipidemia. These results can also be associated with poor health status of Polish population, like in this study. 

In patients with type 2 diabetes hypogonadism is secondary in origin (hypogonadotropic), but possible pathophysiological mechanisms underlying HH in these patients remain partly unexplained. Testosterone in the male can be converted through the action of aromatase to estradiol (E2) and estrone (E1) in the mesenchymal cells, preadipocytes, and adipocytes of adipose tissue. Because obesity is closely connected with diabetes and aging, it has been suggested that excessive E2 secretion due to high aromatase activity in the obese patients may suppress testosterone synthesis as a result of suppression of the hypothalamic secretion of GnRH [10]. But in previous study this hypothesis was not confirmed. In European male aging study estradiol levels in hypogonadal men were significantly lower than in eugonadal men [11], but only few percent of these men were diabetic. In population of T2DM with normal weight Dhindsa et al. observed no association of HH with elevated E2 concentration [8]. Although in all cited studies above testosterone concentrations were inversely related to BMI but low testosterone concentration was not closely depended upon high BMI. In our study, we also observed statistically significant inverse relationship between TT, cfT, and BMI as well as positive correlation between BMI and E2; however, T2DM with HH had significantly higher E2 concentration and lower TT/E2 ratio than eugonadal T2DM. These observations can be in part associated with very high prevalence of obesity in our population (96%) and higher BMI than in other studies (mean BMI 32.4 kg/m^2^). However, Mogri et al. [21] in population of obesity pubertal and postpubertal males showed that obese males had significantly lower TT and cfT concentrations as compared to lean males, but fT concentrations were positively related to age. Interestingly, total and free estradiol levels were significantly lower in males with subnormal testosterone concentrations. So, in this study obesity in young males was associated with low testosterone concentrations, which were not secondary to an increase in estradiol concentrations. In our study we measured total but not free or bioavailable estradiol concentration, while these fractions of estradiol in other studies were directly related to testosterone concentrations in T2DM. 

In previous study, the prevalence of hypogonadism was not dependent on severity of hyperglycemia assessed as glycosylated hemoglobin (HbA1c) levels [1–8, 8–19]. We observed significant negative correlation between HbA1c and testosterone concentration; thus, in population of Polish T2DM severity of hyperglycemia probably can influence the incidence of HH. These findings can also be associated with relative late identification of diabetic patients in our country (mean HbA1c level in all group was 8.4% and in HH patients—8.9%). 

Hypogonadism in men is associated with insulin resistance [5], visceral obesity, the risk of metabolic syndrome [22, 23], vascular complications of diabetes [24], and risk for developing type 2 diabetes [6, 24]. Also in nondiabetic men testosterone levels were inversely associated with insulin levels and HOMA [25]. These observations confirmed the hypothesis that testosterone levels in diabetic men may be influenced by insulin resistance (a key feature of type 2 diabetes) and may play an important role in pathogenesis of HH. 

In mice selective deletion of the insulin receptors from neurons leads to a reduction in LH and FSH levels by 60–90%; testosterone deficiency and disruption of spermatogenesis while incubation of hypothalamic neurons with insulin solution result in the reinforcement of secretion of GnRH [26, 27]. In male mice with deletion of the androgen receptors, increased glucose levels and insulin resistance were observed [28]. Moreover, visceral adiposity is associated with insulin resistance and hypogonadism as a result of testosterone conversion to estradiol [29, 30]. In our study, we observed correlation between testosterone and BMI similar to other studies [18–20]. Thus, appropriate insulin action in the hypothalamo-hypophyseal neurons is crucial in stimulation of testosterone synthesis and spermatogenesis.

Erectile dysfunctions are an important problem in patients with diabetes but also with hypogonadism [18]. We observed significant differences in the degree of erectile dysfunction according to IIEF-5 score between eugonadal patients and men with hypogonadism. Moreover, IIEF-5 score and TT concentrations showed a statistically inverse relationship. 

In the absence of modifiable etiology of hypogonadism and contraindications to treatment, testosterone replacement therapy may be taken into account [13, 30]. Short-term studies in men have shown that testosterone supplementation may improve insulin sensitivity [31–33], but the balance of benefits and risks is still unknown.

A couple of the issues affecting its accuracy can be cited as weaknesses in our data set. First of all our model in no way established a causal link between hypogonadism and type 2 diabetes, because the two conditions might simply overlap and they may have probably separate pathophysiologic pathways.

In Polish population hypogonadism is closely connected with type 2 diabetes and routine testosterone screening should be performed in all T2DM. Our study results strongly justify this practice. The Endocrine Society and the International Society for the Study of the Aging Male (ISSAM) now recommend the measurement of testosterone in patients with type 2 diabetes as a routine basis [13, 30], but this practice is not common and widely accepted in Poland.

In conclusion, we observed high incidence of hypogonadotropic hypogonadism in type 2 diabetic men in Polish population which is associated with poor glycemic control and can be secondary to an increase in estradiol concentrations.

## Figures and Tables

**Table 1:** Characteristics of all T2DM, patients with HH, primary hypogonadism (PH), and with normal testosterone concentration (normal TT/cfT) and nondiabetic control men.

Parameter	T2DM with HH	T2DM with PH	T2DM with normal TT/cfT	All T2DM	P*	Nondiabetic control group	P**
Number of patients	82	5	99	184		149	
Age (years)	56.4 ± 3.2	5.4 ± 3.1	57.6 ± 2.7	58.5 ± 2.3	NS	59.6 ± 3.2	NS
TT (mmol/L)	10.3 ± 1.2	11.1 ± 1.3	16.4 ± 1.95	13.3 ± 1.65	0.002	17.1 ± 1.7	0.001
cfT (nmol/L)	0.225 ± 0.05	0.287 ± 0.06	0.375 ± 0.05	0.315 ± 0.08	0.002	0.382 ± 0.07	0.01
SHBG (nmol/L)	31.3 ± 2.1	30.3 ± 2.6	29.7 ± 2.3	30.9 ± 1.98	0.05	28.7 ± 2.04	0.05
LH (mIU/L)	3.5 ± 0.3	10.3 ± 1.2	7.3 ± 0.3	6.5 ± 0.2	0.02	7.6 ± 0.3	0.02
FSH (mIU/L)	5.8 ± 0.6	13.4 ± 1.8	9.2 ± 0.45	8.8 ± 0.3	0.02	8.6 ± 0.8	NS
Estradiol (pg/mL)	34.5 ± 3.1	32.5 ± 3.7	27.4 ± 3.4	29.4 ± 3.7	0.002	24.5 ± 2.9	0.001
BMI (kg/m^2^)	32.4 ± 1.4	31.1 ± 2.2	30.6 ± 1.3	31.4 ± 0.9	0.05	27.6 ± 1.2	0.02
HbA1c (%)	8.9 ± 1.4	8.8 ± 1.3	8.4 ± 1.2	8.6 ± 1.3	0.002	6.1 ± 0.3	0.002
FPG (mmol/L)	8.3 ± 0.7	8.2 ± 0.6	7.6 ± 0.5	7.9 ± 0.7	0.05	5.3 ± 0.4	0.001

*P shows differences between patients with HH and with normal testosterone levels.

**P shows differences between TD2M and nonobese control group.

Pearson coefficient of BMI and TT: *r* = −0.362; *P* < 0.01 in all T2DM group.

Pearson coefficient of BMI and cfT: *r* = −0.341, *P* < 0.01 in all T2DM group.

Pearson coefficient of BMI and E2: *r* = 0.329; *P* < 0.01 in all T2DM group.

Pearson coefficient of HbA1c and TT: *r* = −0.346, *P* < 0.002 in all T2DM group.

Pearson coefficient of HbA1c and cfT: *r* = −0.336; *P* < 0.005 in all T2DM group.

Pearson coefficient of HbA1c and E2: *r* = 0.337; *P* < 0.002 in all T2DM group.

Pearson coefficient of FPG and TT: *r* = −0.345, *P* < 0.002 in all T2DM group.

**Table 2:** Degree of erectile dysfunction (number of patients; percentage) according to IIEF-5 scale in all T2DM, patients with HH and with normal TT/cfT.

IIEF-5	Men with HH (*n* = 82)	Normal TT/cfT (*n* = 99)	All men (*n* = 184)
Mild	16 (19)	29 (29)	44 (24)
Mild to moderate	28 (34)	29 (29)	58 (31.5)
Moderate	18 (22.5)	22 (23)	45 (24.5)
Severe	20 (24.5)	19 (19)	37 (20)

Significant differences between patients with normal testosterone concentration and patients with hypogonadism (*P* < 0.02).

Variables of IIEF-5 score and TT: *r* = −0.3149, *P* < 0.05.

**Table 3:** Clinical characteristics (number of patients; percentage) of all T2DM, patients with HH and with normal TT/cfT concentration.

Parameter	Men with HH (*n* = 82)	Normal TT/cfT (*n* = 99)	All men (*n* = 184)	*P* value
Obesity	79 (96)	72 (73)	151 (82)	0.002
Current smoker	62 (76)	75 (76)	137 (75)	NS
Hypertension	65 (79)	62 (62)	127 (69)	0.005
Dyslipidemia	61 (74)	65 (65)	126 (69)	0.02
CVD	57 (70)	61 (61)	118 (64)	0.05

*P* value shows significant differences between patients with normal testosterone concentration and patients with hypogonadism.

CVD: cardiovascular disease.
